# Hexaaqua­cobalt(II) bis­(4-amino-3-methyl­benzene­sulfonate)

**DOI:** 10.1107/S1600536809046583

**Published:** 2009-11-11

**Authors:** Wei Zhang, Yuan-Tao Chen

**Affiliations:** a Department of Chemistry, Qinghai Normal University, Xining 810008, People’s Republic of China

## Abstract

In the title mol­ecular salt, [Co(H_2_O)_6_](C_7_H_8_NO_3_S)_2_, the Co^2+^ cation lies on an inversion centre. In the crystal, the components are linked by N—H⋯O and O—H⋯O hydrogen bonds, thereby generating sheets parallel to (001).

## Related literature

For background to hydrogen-bonded networks, see: Tai *et al.* (2007 ▶).
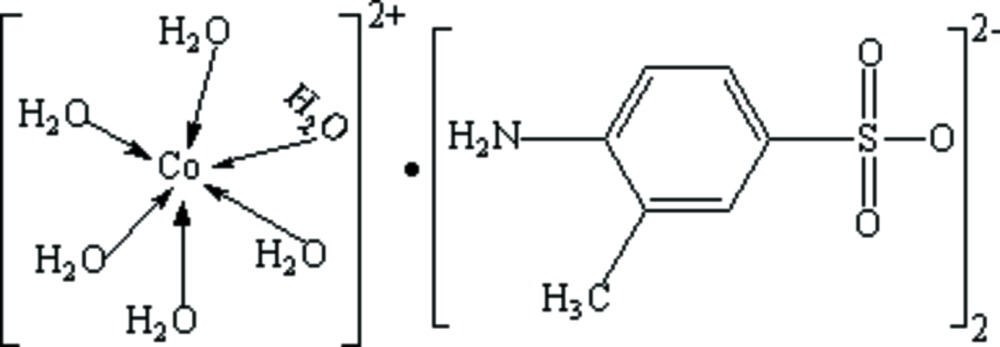


## Experimental

### 

#### Crystal data

[Co(H_2_O)_6_](C_7_H_8_NO_3_S)_2_
*M* *_r_* = 539.43
Monoclinic, 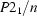

*a* = 6.309 (1) Å
*b* = 7.0513 (11) Å
*c* = 24.262 (4) Å
β = 94.080 (2)°
*V* = 1076.6 (3) Å^3^
*Z* = 2
Mo *K*α radiation
μ = 1.06 mm^−1^
*T* = 293 K
0.21 × 0.16 × 0.12 mm

#### Data collection

Bruker SMART CCD diffractometer
Absorption correction: multi-scan (*SADABS*; Bruker, 2000 ▶) *T*_min_ = 0.809, *T*_max_ = 0.884
5530 measured reflections
1921 independent reflections
1690 reflections with *I* > 2σ(*I*)
*R*_int_ = 0.019

#### Refinement

*R*[*F* ^2^ > 2σ(*F* ^2^)] = 0.025
*wR*(*F* ^2^) = 0.073
*S* = 1.10
1921 reflections
143 parameters
H-atom parameters constrained
Δρ_max_ = 0.42 e Å^−3^
Δρ_min_ = −0.33 e Å^−3^

### 

Data collection: *SMART* (Bruker, 2000 ▶); cell refinement: *SAINT* (Bruker, 2000 ▶); data reduction: *SAINT*; program(s) used to solve structure: *SHELXS97* (Sheldrick, 2008 ▶); program(s) used to refine structure: *SHELXL97* (Sheldrick, 2008 ▶); molecular graphics: *SHELXTL* (Sheldrick, 2008 ▶); software used to prepare material for publication: *SHELXTL*.

## Supplementary Material

Crystal structure: contains datablocks global, I. DOI: 10.1107/S1600536809046583/hb5188sup1.cif

Structure factors: contains datablocks I. DOI: 10.1107/S1600536809046583/hb5188Isup2.hkl

Additional supplementary materials:  crystallographic information; 3D view; checkCIF report

## Figures and Tables

**Table 1:** Selected bond lengths (Å)

Co1—O6	2.0515 (14)
Co1—O4	2.0866 (13)
Co1—O5	2.0868 (13)

**Table 2:** Hydrogen-bond geometry (Å, °)

*D*—H⋯*A*	*D*—H	H⋯*A*	*D*⋯*A*	*D*—H⋯*A*
N1—H1*A*⋯O2^i^	0.86	2.47	3.214 (2)	145
N1—H1*B*⋯O4	0.86	2.56	3.129 (2)	125
O4—H7⋯O2^i^	0.85	2.00	2.8300 (19)	167
O4—H8⋯O3^ii^	0.85	1.92	2.7675 (19)	176
O5—H9⋯O1^iii^	0.85	1.94	2.7828 (19)	170
O5—H10⋯O3^iv^	0.85	1.95	2.7963 (19)	174
O6—H11⋯O1^iv^	0.85	1.93	2.7711 (19)	169
O6—H12⋯O2^ii^	0.85	1.90	2.7419 (19)	173

Symmetry codes: (i) 
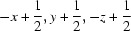
; (ii) 
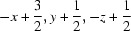
; (iii) 
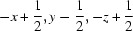
; (iv) 
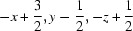
.

**Fig. 1.**
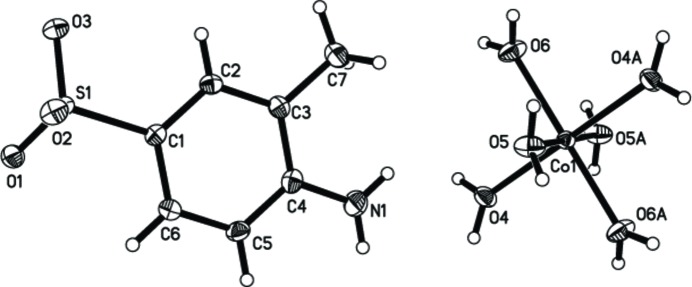
The molecular structure of (I) showing 30% displacement ellipsoids. Atosm with suffix A are generated by the symmetry operation (1–x, 1–y, –z).

## supplementary crystallographic information

### Experimental

A solution of 1.0 mmol 4-amino-3-methyl-benzenesulfonic acid and 1.0 mmol NaOH in 10 ml ethanol was added to a solution of 0.5 mmol Co(CH_3_COO)_2_4H_2_O in 5 ml e thanol at room temperature. The mixture was refluxed for 4 h with stirring, then the resulting precipitate was filtered, washed, and dried *in vacuo* over P_4_O_10_ for 48 h. Pink blocks of (I) were obtained by slowly evaporating from methanol at room temperature.

### Crystal data

**Table:**

[Co(H_2_O)_6_](C_7_H_8_NO_3_S)_2_	*F*(000) = 562
*M**_r_* = 539.43	*D*_x_ = 1.664 Mg m^−^^3^
Monoclinic, *P*2_1_/*n*	Mo *K*α radiation, λ = 0.71073 Å
Hall symbol: -P 2yn	Cell parameters from 3103 reflections
*a* = 6.309 (1) Å	θ = 3.3–28.3°
*b* = 7.0513 (11) Å	µ = 1.06 mm^−^^1^
*c* = 24.262 (4) Å	*T* = 293 K
β = 94.080 (2)°	Block, pink
*V* = 1076.6 (3) Å^3^	0.21 × 0.16 × 0.12 mm
*Z* = 2	

### Data collection

**Table:**

Bruker SMART CCD diffractometer	1921 independent reflections
Radiation source: fine-focus sealed tube	1690 reflections with *I* > 2σ(*I*)
graphite	*R*_int_ = 0.019
φ and ω scans	θ_max_ = 25.1°, θ_min_ = 1.7°
Absorption correction: multi-scan (*SADABS*; Bruker, 2000)	*h* = −7→7
*T*_min_ = 0.809, *T*_max_ = 0.884	*k* = −8→6
5530 measured reflections	*l* = −25→28

### Refinement

**Table:**

Refinement on *F*^2^	Secondary atom site location: difference Fourier map
Least-squares matrix: full	Hydrogen site location: inferred from neighbouring sites
*R*[*F*^2^ > 2σ(*F*^2^)] = 0.025	H-atom parameters constrained
*wR*(*F*^2^) = 0.073	*w* = 1/[σ^2^(*F*_o_^2^) + (0.035*P*)^2^ + 0.4723*P*] where *P* = (*F*_o_^2^ + 2*F*_c_^2^)/3
*S* = 1.10	(Δ/σ)_max_ < 0.001
1921 reflections	Δρ_max_ = 0.42 e Å^−^^3^
143 parameters	Δρ_min_ = −0.32 e Å^−^^3^
0 restraints	Extinction correction: *SHELXL97* (Sheldrick, 2008), Fc^*^=kFc[1+0.001xFc^2^λ^3^/sin(2θ)]^-1/4^
Primary atom site location: structure-invariant direct methods	Extinction coefficient: 0.0268 (16)

### Special details

**Table:**

Geometry. All e.s.d.'s (except the e.s.d. in the dihedral angle between two l.s. planes) are estimated using the full covariance matrix. The cell e.s.d.'s are taken into account individually in the estimation of e.s.d.'s in distances, angles and torsion angles; correlations between e.s.d.'s in cell parameters are only used when they are defined by crystal symmetry. An approximate (isotropic) treatment of cell e.s.d.'s is used for estimating e.s.d.'s involving l.s. planes.
Refinement. Refinement of *F*^2^ against ALL reflections. The weighted *R*- factor *wR* and goodness of fit *S* are based on *F*^2^, conventional *R*-factors *R* are based on *F*, with *F* set to zero for negative *F*^2^. The threshold expression of *F*^2^ > σ(*F*^2^) is used only for calculating *R*-factors(gt) *etc*. and is not relevant to the choice of reflections for refinement. *R*-factors based on *F*^2^ are statistically about twice as large as those based on *F*, and *R*- factors based on ALL data will be even larger.

### Fractional atomic coordinates and isotropic or equivalent isotropic displacement parameters (Å^2^)

**Table:**

	*x*	*y*	*z*	*U*_iso_*/*U*_eq_	
Co1	0.5000	0.5000	0.0000	0.02153 (14)	
S1	0.59383 (7)	0.51047 (6)	0.403529 (19)	0.02233 (15)	
O1	0.5037 (2)	0.66812 (19)	0.43304 (5)	0.0324 (3)	
O2	0.5014 (2)	0.32921 (19)	0.41856 (5)	0.0315 (3)	
O3	0.8256 (2)	0.50668 (17)	0.41067 (6)	0.0311 (3)	
O4	0.3989 (2)	0.73519 (19)	0.04326 (6)	0.0363 (3)	
H7	0.2775	0.7762	0.0506	0.054*	
H8	0.4888	0.8156	0.0567	0.054*	
O5	0.4048 (2)	0.3100 (2)	0.05939 (6)	0.0365 (4)	
H9	0.2842	0.2560	0.0589	0.055*	
H10	0.4924	0.2201	0.0664	0.055*	
O6	0.7970 (2)	0.50949 (18)	0.04025 (7)	0.0418 (4)	
H12	0.8684	0.6054	0.0519	0.063*	
H11	0.8706	0.4134	0.0507	0.063*	
N1	0.3463 (3)	0.6380 (3)	0.16739 (7)	0.0474 (5)	
H1A	0.2229	0.6845	0.1580	0.057*	
H1B	0.4320	0.6115	0.1425	0.057*	
C1	0.5244 (3)	0.5467 (2)	0.33285 (7)	0.0232 (4)	
C2	0.6647 (3)	0.5036 (2)	0.29306 (8)	0.0247 (4)	
H2	0.7980	0.4545	0.3039	0.030*	
C3	0.6088 (3)	0.5326 (2)	0.23743 (8)	0.0260 (4)	
C4	0.4060 (3)	0.6062 (3)	0.22179 (8)	0.0289 (4)	
C5	0.2676 (3)	0.6489 (3)	0.26221 (8)	0.0307 (4)	
H5	0.1341	0.6985	0.2518	0.037*	
C6	0.3248 (3)	0.6188 (3)	0.31715 (8)	0.0289 (4)	
H6	0.2301	0.6467	0.3437	0.035*	
C7	0.7578 (4)	0.4865 (3)	0.19380 (9)	0.0360 (5)	
H7A	0.8888	0.4387	0.2110	0.054*	
H7B	0.7854	0.5991	0.1733	0.054*	
H7C	0.6945	0.3923	0.1693	0.054*	

### Atomic displacement parameters (Å^2^)

**Table:**

	*U*^11^	*U*^22^	*U*^33^	*U*^12^	*U*^13^	*U*^23^
Co1	0.0198 (2)	0.0217 (2)	0.0232 (2)	−0.00091 (13)	0.00196 (14)	−0.00005 (12)
S1	0.0203 (3)	0.0220 (3)	0.0245 (3)	−0.00008 (16)	−0.00023 (18)	0.00040 (16)
O1	0.0316 (7)	0.0326 (7)	0.0328 (7)	0.0041 (6)	0.0005 (6)	−0.0082 (6)
O2	0.0299 (7)	0.0284 (7)	0.0359 (8)	−0.0036 (6)	0.0002 (6)	0.0071 (6)
O3	0.0220 (7)	0.0343 (8)	0.0362 (8)	0.0004 (5)	−0.0024 (6)	0.0012 (5)
O4	0.0278 (7)	0.0340 (8)	0.0476 (9)	−0.0019 (6)	0.0072 (6)	−0.0164 (6)
O5	0.0270 (7)	0.0361 (8)	0.0474 (9)	0.0027 (6)	0.0088 (6)	0.0160 (7)
O6	0.0311 (8)	0.0281 (8)	0.0633 (11)	−0.0004 (6)	−0.0171 (7)	−0.0025 (6)
N1	0.0528 (12)	0.0596 (13)	0.0288 (9)	0.0205 (10)	−0.0043 (8)	0.0047 (9)
C1	0.0247 (9)	0.0198 (8)	0.0248 (9)	−0.0011 (7)	−0.0006 (7)	0.0021 (7)
C2	0.0236 (9)	0.0197 (9)	0.0307 (10)	0.0007 (7)	0.0009 (8)	0.0008 (7)
C3	0.0304 (10)	0.0196 (9)	0.0283 (10)	−0.0003 (7)	0.0041 (8)	−0.0009 (7)
C4	0.0375 (11)	0.0222 (9)	0.0263 (10)	0.0008 (8)	−0.0019 (8)	0.0025 (7)
C5	0.0268 (10)	0.0306 (10)	0.0338 (11)	0.0077 (8)	−0.0042 (8)	0.0003 (8)
C6	0.0261 (10)	0.0307 (10)	0.0299 (10)	0.0031 (8)	0.0031 (8)	−0.0011 (8)
C7	0.0418 (12)	0.0335 (11)	0.0334 (11)	0.0050 (9)	0.0085 (10)	0.0004 (8)

### Geometric parameters (Å, °)

**Table:**

Co1—O6^i^	2.0515 (14)	N1—C4	1.365 (2)
Co1—O6	2.0515 (14)	N1—H1A	0.8600
Co1—O4^i^	2.0866 (13)	N1—H1B	0.8600
Co1—O4	2.0866 (13)	C1—C6	1.386 (2)
Co1—O5	2.0868 (13)	C1—C2	1.389 (3)
Co1—O5^i^	2.0868 (13)	C2—C3	1.386 (3)
S1—O1	1.4593 (13)	C2—H2	0.9300
S1—O3	1.4603 (14)	C3—C4	1.407 (3)
S1—O2	1.4617 (13)	C3—C7	1.500 (3)
S1—C1	1.7580 (18)	C4—C5	1.392 (3)
O4—H7	0.8498	C5—C6	1.372 (3)
O4—H8	0.8498	C5—H5	0.9300
O5—H9	0.8500	C6—H6	0.9300
O5—H10	0.8499	C7—H7A	0.9600
O6—H12	0.8499	C7—H7B	0.9600
O6—H11	0.8499	C7—H7C	0.9600
			
O6^i^—Co1—O6	180.0	H12—O6—H11	105.7
O6^i^—Co1—O4^i^	92.07 (6)	C4—N1—H1A	120.0
O6—Co1—O4^i^	87.93 (6)	C4—N1—H1B	120.0
O6^i^—Co1—O4	87.93 (6)	H1A—N1—H1B	120.0
O6—Co1—O4	92.07 (6)	C6—C1—C2	120.05 (17)
O4^i^—Co1—O4	180.0	C6—C1—S1	118.67 (14)
O6^i^—Co1—O5	90.56 (6)	C2—C1—S1	121.28 (14)
O6—Co1—O5	89.44 (6)	C3—C2—C1	120.94 (17)
O4^i^—Co1—O5	87.15 (6)	C3—C2—H2	119.5
O4—Co1—O5	92.85 (6)	C1—C2—H2	119.5
O6^i^—Co1—O5^i^	89.44 (6)	C2—C3—C4	118.72 (17)
O6—Co1—O5^i^	90.56 (6)	C2—C3—C7	121.77 (18)
O4^i^—Co1—O5^i^	92.85 (6)	C4—C3—C7	119.51 (17)
O4—Co1—O5^i^	87.15 (6)	N1—C4—C5	120.13 (17)
O5—Co1—O5^i^	180.0	N1—C4—C3	120.29 (18)
O1—S1—O3	112.18 (8)	C5—C4—C3	119.58 (17)
O1—S1—O2	111.56 (9)	C6—C5—C4	121.08 (17)
O3—S1—O2	111.60 (7)	C6—C5—H5	119.5
O1—S1—C1	106.81 (8)	C4—C5—H5	119.5
O3—S1—C1	107.24 (8)	C5—C6—C1	119.63 (17)
O2—S1—C1	107.10 (8)	C5—C6—H6	120.2
Co1—O4—H7	133.5	C1—C6—H6	120.2
Co1—O4—H8	120.2	C3—C7—H7A	109.5
H7—O4—H8	106.2	C3—C7—H7B	109.5
Co1—O5—H9	125.3	H7A—C7—H7B	109.5
Co1—O5—H10	113.7	C3—C7—H7C	109.5
H9—O5—H10	103.7	H7A—C7—H7C	109.5
Co1—O6—H12	129.0	H7B—C7—H7C	109.5
Co1—O6—H11	125.2		
			
O1—S1—C1—C6	−38.02 (17)	C2—C3—C4—N1	179.47 (17)
O3—S1—C1—C6	−158.47 (14)	C7—C3—C4—N1	−0.9 (3)
O2—S1—C1—C6	81.62 (16)	C2—C3—C4—C5	0.3 (3)
O1—S1—C1—C2	142.30 (14)	C7—C3—C4—C5	179.96 (17)
O3—S1—C1—C2	21.85 (16)	N1—C4—C5—C6	−179.70 (19)
O2—S1—C1—C2	−98.06 (15)	C3—C4—C5—C6	−0.6 (3)
C6—C1—C2—C3	0.4 (3)	C4—C5—C6—C1	0.7 (3)
S1—C1—C2—C3	−179.97 (13)	C2—C1—C6—C5	−0.6 (3)
C1—C2—C3—C4	−0.2 (3)	S1—C1—C6—C5	179.75 (15)
C1—C2—C3—C7	−179.85 (17)		

Symmetry codes: (i) −*x*+1, −*y*+1, −*z*.

### Hydrogen-bond geometry (Å, °)

**Table:**

*D*—H···*A*	*D*—H	H···*A*	*D*···*A*	*D*—H···*A*
N1—H1A···O2^ii^	0.86	2.47	3.214 (2)	145
N1—H1B···O4	0.86	2.56	3.129 (2)	125
O4—H7···O2^ii^	0.85	2.00	2.8300 (19)	167
O4—H8···O3^iii^	0.85	1.92	2.7675 (19)	176
O5—H9···O1^iv^	0.85	1.94	2.7828 (19)	170
O5—H10···O3^v^	0.85	1.95	2.7963 (19)	174
O6—H11···O1^v^	0.85	1.93	2.7711 (19)	169
O6—H12···O2^iii^	0.85	1.90	2.7419 (19)	173

Symmetry codes: (ii) −*x*+1/2, *y*+1/2, −*z*+1/2; (iii) −*x*+3/2, *y*+1/2, −*z*+1/2; (iv) −*x*+1/2, *y*−1/2, −*z*+1/2; (v) −*x*+3/2, *y*−1/2, −*z*+1/2.
